# Supplementary material for: A new phylodynamic model of Mycobacterium bovis transmission in a multi-host system uncovers the role of the unobserved reservoir
Source: PLoS Comput Biol. 2021 Jun 25;17(6):e1009005. doi: 10.1371/journal.pcbi.1009005 (PMC8266114; doi:10.1371/journal.pcbi.1009005)
Supplement: S1 Table — Minimum diversity model (Min), Intermediate diversity model (Int), Maximum diversity model (Max), A single reservoir connecting every farm (Giant), No Connecting Reservoirs (None). (PDF) [file pcbi.1009005.s006.pdf]

S1 Table. **Comparison of the mean posterior values for the parameters in each of the 5 models:** Minimum diversity model (Min), Intermediate diversity model (Int), Maximum diversity model (Max), A single reservoir connecting every farm (Giant), No Connecting Reservoirs (None).

	Min	Giant	Max	Int	None
AIC	2192.7	3056.8	3232.2	3816.2	4370.8
β (per contact per day)	6.31×10^{-6}	4.46×10^{-6}	4.11×10^{-6}	4.71×10^{-6}	7.08×10^{-6}
σ (per day)	0.042	0.042	0.051	0.057	0.014
γ (per day)	0.0062	0.0068	0.0042	0.0058	0.0075
β_{CR} (per contact per day)	2.45×10^{-6}	5.15×10^{-6}	2.78×10^{-6}	2.57×10^{-6}	3.02×10^{-6}
β_{RC} (per contact per day)	4.39×10^{-6}	4.29×10^{-6}	4.57×10^{-6}	1.84×10^{-6}	1.89×10^{-6}
Ω	0.51	0.55	0.48	0.63	0.57
μ (per day)	0.001	0.0025	0.0029	0.0014	0.0010
