# Supplementary material for: A new phylodynamic model of Mycobacterium bovis transmission in a multi-host system uncovers the role of the unobserved reservoir
Source: PLoS Comput Biol. 2021 Jun 25;17(6):e1009005. doi: 10.1371/journal.pcbi.1009005 (PMC8266114; doi:10.1371/journal.pcbi.1009005)
Supplement: Attachment — Submitted filename: Plos CB_reply OHare et al FINAL.docx [file pcbi.1009005.s007.docx]

Dear Editor,

Thank you very much for the opportunity to submit a revised version of this manuscript. As required, we submit two copies of this paper (one with highlighted changes) and this detailed response. We trust that we have done so comprehensively and await your further reply.

With best regards,

Rowland Kao (for all authors).

**Response.**

We thank the three referees for their comments regarding the interest and importance of our system, and the appropriateness of our methodology, and inferred suitability for PLoS Computational Biology. We outline in detail our point by point response below. We note here that, throughout the text, the revisions outlined in detail below have resulted in a larger number of text edits to improve the flow of the manuscript. We have not highlighted all these in yellow as they are not directly responding to the referees’ comments and therefore in our view would only confuse the reviewers.

Detailed response.

Reviewer's Responses to Questions

**Comments to the Authors:
Please note here if the review is uploaded as an attachment.**

Reviewer #1: I really enjoyed reading this paper. The authors take a novel computational approach to assessing the potential role of an unsampled reservoir population in creating the observed SNP diversity of Mycobacterium bovis in cattle. I support this paper for eventual publication but found that there were a number of components that needed more details.

*Response: We thank the reviewer for these positive comments.*

1. The authors provide details on the model structure as text. I don't think this is sufficient to be able to repeat any of their analyses or build off of their work here and easily apply this work to another system. Providing all, or some key parts, of the code would address this. I also wonder if you could include a mock figure indicating where SNPs are generated in the different scenarios.

*Response. In response to this recommendation, we have added a new diagram (figure 4 in the supporting information) and also further details of the modelling approach. We also include a link to a github site, where all code will be available.*

2. The likelihood only addresses the SNP data, but not any of the epidemiological data. Yet the authors clearly have the movement information and whether positive cattle herds are linked by a prior movement event or not, which would be indicative of a wildlife reservoir as well. The authors mention this around line 273, but I think more could be said here. Given what the authors have accomplished here, what are the pros/cons and difficulties that would be involved in trying to also incorporate more of the epidemiological data.

*Response. We agree that availability of the epidemiological data would be helpful in recreating and understanding our analysis. While the terms of our confidentiality agreements do not allow us to disclose the exact patterns, included in the github respository are the data files which would allow a recreation of the distributions we use to generate the model. We have indicated that a clear next step would be the estimation of parameters, using the combined epidemiological and genetic information which we agree with the reviewer would be a very important addition that we aim to do next. We note that we do not think it necessarily difficult – it manly requires a reassessment of the metric to incorporate key features of the epidemiological signature – but the additional analysis of the epidemiological data to develop and test alternative more detailed metrics would require more time.*

3. Figure 6 illustrates how some of the posteriors are bumping up against the edges of the uniform prior distributions. This warrants some discussion, if not a complete re-run with wider priors on some of these. This may be an issue of computational time, but that also wasn't well described.

*Response: We agree with reviewer 1 that the some of the posteriors in figure 6 are close to the edge of the prior distributions used. However, these priors are taken from known field and experimental values and therefore limited by the available biological knowledge. We have added a note to emphasize this in the figure caption. In the case of sigma in particular, there is quite a wide range in the value of this transition rate and our modelling suggests that the length of time an animal is in this stage is towards the longer estimate. We also make this note in the text as well (line 232)*

4. I needed more help relating some of the modeling scenarios to biological systems. For example, how would you have a wildlife reservoir that does not generate any SNP diversity (the best model of low diversity). This would seem to imply no transmission within the wildlife species and all transmission is contained in the spillover process. If true, then I'm not sure reservoir is the right term for the wildlife population. Could these scenarios be related to Haydon et al 2002's circles and squares diagram? And does this scenario imply that R0 is less than one in the badger population alone, otherwise one would accrue SNP diversity in the wildlife population? How does this conclusion match with the other badger-TB work?

*Response: The reviewer 1 is correct that the ‘no SNP’ generation scenario is a best an approximation of the likely true situation. This scenario represents our approach to modelling circumstances where the wildlife reservoir does not “hold” infection for long enough to generate substantial genetic diversity on its own – rather the persistence is based on regular re-introductions from the cattle population. As substitution rates are very slow (on the order of half a year per SNP) even if the reservoir ‘held’ infection for say, 3 months (i.e. the minimum time it takes for a herd to clear restrictions and be considered free of Tb) it would not likely generate much diversity, with a low probability of generating new SNPs. While a more detailed model would have allowed explicit consideration of the reservoir having this kind of role, our choice of models was made to allow us to contrast two extreme scenarios. We note that our result implies that if badgers are involved in the reservoir, it also implies they only play a relatively small role for these data. In more recent work, an ongoing intensive effort to examine the impact of badger controls on cattle Tb in Northern Ireland; a preprint of this work has recently been put in the public domain -* <https://doi.org/10.1101/2021.03.12.435101>. *For ease of reference, I include here a figure with the outcome of an analysis using the phylodynamic inference software ‘BASTA’ (de Maio et al. PLOS Computational Biology 14:e1006117.* [*https://doi.org/10.1371/journal.pcbi.1006117*](https://doi.org/10.1371/journal.pcbi.1006117)*) that provides corroborating evidence showing that the probability of badger-to-cattle transition is likely substantially lower than cattle-to-cattle (or in the figure, ‘bovine-to-bovine’). A similar analysis for East Cumbria in the north east of England similarly provided low (but non-zero) estimates for the transition rates from badger-to-cattle, albeit in an emergent infection scenario (doi:* [*https://doi.org/10.1101/2020.11.12.379297*](https://doi.org/10.1101/2020.11.12.379297)*). Both these analyses suggest that our outcome (achieved without the badger samples) is plausible.*

Minor points.
Spell out Northern Ireland for those less familiar with that region of the world.

*Response: Done as recommended*

I was confused by the legend on Fig. 3. It states that R0 <1 indicates the proportion of the posterior where the removal of the external force would result in disease eradication. I would have thought that if R0 <1 then that indicates eradication without any need for additional removal of the external force of infection. Maybe this gets to an earlier point about whether badgers maintain the disease in the absence of cattle.

*Response: We have amended the text to make it clear that this is the value of R0 in the absence of the reservoir, as an indicator of the importance of the test-and-slaughter programme in controlling bTB in Northern Ireland. The figure has also been moved to supplementary information in response to a comment from reviewer 3.*

Reviewer #2: In the paper "A new phylodynamic model of Mycobacterium bovis transmission in a multi-host system uncovers the role of the unobserved reservoir" , O'hare et al use mathemathical compartment modeling to explore how hidden reservoirs contribute to the observed bTb circulation in Northern Ireland. It is an interesting and well-written paper that deals with a hot topic - The role of badgers in bTb spread. (And could therefore be used in this ongoing debate about the notorious control work that has been done in the UK). The paper concludes that the best-fitting model (among the 5 models tested) is one that ascribes a central role to both cattle-to-cattle transmission but with the addition of a hidden reservoir that can infect local farms. Interestingly, the paper offers no data to support the idea that this hidden reservoir consists of badgers, although it is heavily implied.

*Response: We thank the reviewer for these positive comments. The reviewer is correct to point out that we are careful to avoid directly attributing the role of the reservoir to badgers, though we do note the reasons why we believe it is consistent with badger involvement (we’ve included more details on this in the reply to the next comment).*

Below follows some specific comments to the paper:
- The link to badgers is not clear. Although badgers are not mentioned in the title, they are repeatedly mentioned in both the abstract and the main text. It is mentioned in the "Data" chapter that whole-genome sequencing of roadkilled badgers have been undertaken, although these data were not used to inform the simulations. Why not? Badger sequences also appear in Figure 1, and from this figure it is clear that at least some badger-originating sequences are closely related to cattle. I worry that this paper will be seen as demonstrating the role of badgers specifically, and while the hidden reservoir may indeed consist of badgers, there are many other plausible explanations, some of which the authors themselves discuss, like untracked movements, shared pastures, bulls etc.

*Response: The reviewer has correctly noted the difficult balance we are aiming to achieve for what is a very controversial subject in Britain and Ireland. Given the available evidence in other scenarios where bacterial WGS has shown badgers can have a substantial involvement (Crispell et al. 2019), not mentioning badgers at all would stretch the bounds of believability. We do note the reviewer’s caution and, while in our view we cannot avoid the question of badgers completely, we have revised the text, restricting this to the introduction and discussion and changing ‘wildlife reservoir’ to ‘hidden reservoir’ throughout. Additional text in the discussion is included to indicate why we think it likely that the reservoir is at least associated with badgers.*

*We do not use the available badger samples (6 in total) in this particular analysis, other than to note the phylogenetic proximity, as our aim is to emphasize the outcome that, even with a very simple model for the hidden reservoir with no samples at all, we can make clear model selection choices, thereby providing insight into the characteristics of that reservoir (also they are so few in number compared to the cattle isolates, they will likely have little influence on the inference).*

*These data biases are typical for Great Britain and Northern Ireland, as badger data are available in only very limited locations. As we noted in the response to reviewer 1, there are independent estimates in Northern Ireland, in a location where there are many badger-derived genomes, and phylodynamic analyses have come to similar conclusions.*

- Figure 1 - It is unclear to me why this figure is in the paper. It is taken directly from Trewby et al, 2015, and does not seem particularly relevant for the simulations except to demonstrate the basis for the SNP distribution.

*Response: the reviewer is correct; this is a duplicate of the figure in Trewby et al. It is there for the convenience of the reader, who we thought would likely prefer to have the phylogeny presented here, rather than having to look at another paper. However, if the reviewer remains of this view upon scrutiny of the revised paper, we are not adverse to removing it.*

- I'm not 100% sure I understand the SETI model on page 4. Is there a separate process that seeds infection to the reservoir and the reservoirs are modeled with separate ODEs? I assume there's also a process for culling of infected that's not specified in this model? On page 6 movement restrictions upon a positive test is described, but it says nothing about test-positive animals being culled.

*Response: We now include text to make it clear that our model is chosen to be a ‘minimalist’ extension of the SETI model to consider the reservoir, without requiring substantial greater model complexity, or extra parameters, to do it. There are of course limitations to our approach, but its key strength is that it allows us to distinguish between spatial and non-spatial reservoirs, and contributions of the reservoir to pathogen diversity. In the study area over this period, no badger culling was in operation and therefore the reservoir is not perturbed in our model. We now clarify this in the text.*

- On page 6-7, the 5 different simulation setups are described. It would be helpful if the authors could use this terminology when they refer to specific scenarios elsewhere in the paper (e.g. 3A). In fact I am confused as to which model ended up as the winner in the end. On page 8 the most parsimonious is the model where "that generated NO additional diversity within the reservoir population 3". In the table on the following page (with AIC scores), a low-diversity model is prefered. Later on the same page the best model is one that "does not generate substantial new genetic diversity", while again on page 10 "our preferred model incorporates a reservoir which generates NO independent diversity".

*Response: we have tightened up the language throughout in response to the reviewer’s recommendations and also provide more details about the individual models. We use the term ‘minimal diversity’ throughout to indicate that, while diversity is generated (by the badgers) it will generate none via the reservoir.*

- The mutation rate prior and posterior seem extremely high for bTb. In my experience they are much closer to the 1.0E-7 mark if we are talking about the whole-genome rate. Is this rate for some smaller set, eg just the complement of SNP sites?

*Response: We apologise for the confusion here: we had missed putting in the units of the substitution rate. It is in SNPs generated per day to match the units used for the remaining parameters. We also had a typo in the text (an extra zero) that is now corrected. Our prior estimates were based on the previous estimate in this system (an upper limit of less than 1 substitution every two years from Trewby et al. 2015).*

- On the very last sentence of the results is an inverted question mark. - Should be a "greater than" sign?

*Response: this typo has now been fixed (blame overleaf ☺ ).*

- I can understand that putting potentially identifiable movement data online can be impossible under EU law. Would the authors at least consider sharing the code they used in their simulations?

*Response: We wholeheartedly agree and it was an oversight on our part not to have included this in the first submission. We have now added a link to a github site with all available code and the relevant data files.*

- Figure 6 - The scale of sigma is too large to see anything. What is the unit of sigma? This might be obvious from table 2 but I can't figure it out. (0.042 = 23.8 days ?)

*Response: we have increased the font sizes and added in the units as recommended.*

Reviewer #3: In this work, authors present a coupled dynamical model where the dynamics of bovine tuberculosis (bTB) transmission through cattle in Northern Ireland, between 1995 and 2010 is described. The model integrates three dynamical processes (namely, bTB dynamics, cattle movement between farms and genetic diversity dynamics in the pathogen); and its main objective is to provide a modeling framework through which the genetic diversity patterns that are observed among the registered outbreaks across the territory during the mentioned period can be explained only as a consequence of incorporating to the models the interaction between herds and wildlife reservoirs that are carefully modelled, in this case Eurasian badgers.

I think the work is appealing, and the modeling framework presented is comprehensive, and a valuable tool to understand the contribution of wild reservoirs to the control of this important economical and public health problem.

*Response: We thank the reviewer for these positive comments about the framework and the importance of the underlying question.*

I have, however a series of specific concerns that are mostly related to the way that the model is described (which I think, in some aspects is a bit cumbersome, and/or leaves important questions unaddressed), as well as the way some of the results are presented. If authors managed to provide an answer to these questions, I would be happy to recommend their work for publication in the journal.

*Response: We have taken aboard reviewer 3’s concerns and have substantially altered the relevant text throughout including a new figure to address some of the recommendations. Where appropriate, we respond in more detail below.*

Methods: the explanation of the algorithm provided in the text within the methods section is quite cumbersome at times. I would suggest authors to try to structure more explicitly the information about the three dynamical processes coupled in their model (i.e. infection dynamics, strains evolution and cattle movements). Similarly, I strongly suggest authors to include a global algorithm summary as well.

*Response: We have added paragraph headings to help structure the Methods section and made several edits to the text to make the explanation clearer. By moving the description of the Gillespie algorithm to the top of this section we expect that the other paragraphs now have a better context within the model. We agree that a summary of the algorithm would be useful and have included a flow diagram of our algorithm in Figure 4.*

2. In particular, I found confusing the description of how do the model does two things that are tightly related to each other, that is, keeping track of the total amount of susceptible individuals, as well as the total amount of individuals in each herd. Specifically, it would seem that authors introduce, at each time step, corrections in the population of each herd, to make the evolution of herd sizes compatible to the data. However, I see that this can be done in at least three ways, and it is not clear to me which one authors used:

a. The total amount of susceptible animals during the simulations is in fact the variable whose time evolution is to be “fitted” to the data of herd size, neglecting the contribution to herd size of infected animals. Therefore, the animals added/removed to simulate births/deaths are all susceptible.
b. The total amount of animals, both susceptible and infected, during the simulations is in fact the variable whose time evolution is to be “fitted” to the data of herd size. Here, the animals added/removed to simulate births/deaths could be:
b.1 all susceptible.
b.2 Distributed between infection classes proportionally to their prevalences in each herd, at each time.

My understanding is that authors chose option a, but I cannot be sure, reading the text. In any case, they should state explicitly how this was done, and, if their choice implies neglecting the contribution of infected individuals to the total herd size, it would be helpful to see, for example, the distribution of the fraction of infected individuals across herds/and or time, which would be very helpful for the reader to get a good grasp of how sensible that simplification may be.

*Response: We set the herd size from the known distribution of herd sizes in our dataset and keep this fixed throughout the simulation assuming that animals moved off a farm (to other farms or to slaughter) are replaced with births which are susceptible. We note in the data section that the herd sizes were not seen to substantially change over time (lines 51-53). Our initial approach did include explicit modelling of susceptible animals, herd size, births, and deaths and gave similar results to the model described in the paper, however run times were very long. Removing this explicit approach resulted in substantially more efficient simulations.*

3. I miss, also in the methods section, a diagram reuniting the info of how many animals, herds, movements between herds, herd breakdowns, isolates, SNPs, simulations, reservoirs, etc. are being simulated.

*Response: These have now been included in the additional figure 4. Most of the figures the reviewer requests are drawn from distributions informed from the data or obtained as a result of our simulations. We have included as much information in this figure as possible. The remainder of the relevant information about the distributions can be found by downloading the relevant files from the github site (these should be easily interpreted with the information provided)*

4. How is the movement distribution defined? The usage of the word distribution seems to imply that authors built a frequency distribution of movements as a function, for example, of the distance between farms, and draw movements from this distribution stochastically, in their simulations. This would allow movements among all farms, including those that were not involved in cattle exchange according to the data. Is this correct? It could also be that movement frequencies (propensities) are proportional to some other variable, such as herd sizes of receptor and/or sender herds, or proportional to the frequency of trade of each farm as observed in the dataset. In any case, how are movement propensities written down within the Gillespie algorithm? Do they allow connecting farms for which no cattle movement was recorded in the dataset, via cattle movements during the simulations?

*Response: In response to the reviewer’s report, we have reworked the description of the movement algorithm. We now describe in the updated text that the observed number of animals moved in each fortnightly step were very stable over the time period we simulated (we attach the distribution below). Our movement data contained a record of the number of times pairs of farms traded and the number of animals traded in each trade. At the end of each time step, after we have updated the disease states of each animal, we sample from the known distribution of farm-farm movements until we have moved the required number of animals for that period (19,108 animals moved in each step).*

5. I have two doubts concerning the assignment of reservoirs to herds according to model 3:

5.1 In lines 43-44, we read that data anonymization implies scaling of distances (plus rotation and translation). How is it possible, then, to know which farms are within a 4 km range in order to decide whether it makes sense or not to assign them a single badger reservoir?

*Response: We received an anonymised set of farms and their locations from the data holders but we asked for this to include a list of all those farms that were with 4km of each other before applying the transformation. Figure 5 shows the transformed locations with those that we know are less than 4km apart. We have added a note to this effect to the figure.*

5.2 In lines 102-109 we read that no shared reservoir was included for the farms without movements. If that is the case (especially if the answer to the last question in point 4 is positive), this would introduce a bias in the simulations, which would generate more disease prevalence in the farms for which we had location data, bc they, by construction, can share reservoirs. An easy, alternative null model for these farms where location data is unavailable would be that the proportion of farm groups of size=2,3, etc. sharing a common reservoir should match that of the data for which we do have farms´ locations. If authors think this should no introduce substantial modifications, I think this should be discussed, and properly justified.

*Response: In response to the reviewer’s question, we have repeated the simulations with dummy locations assigned to all herds, by assuming that the herds for which we have locations, are a random subset of all herds and preserving the distance relationships that are observed. This model did not converge after 750 iterations (substantially more attempted than for the other models considered). We describe this in brief in the paper, and offer a plausible explanation – statistical analyses have previously shown that the most important predictor of a herd having a breakdown/outbreak of Tb, is a previous history of breakdowns (see Karolemeas et al. 2011 or Bessell et al. 2012 for example). This implies that there is a considerable difference in risk across herds that is not captured by simple herd demographic data available to us. This therefore provides a plausible rationale for our outcome – in restricting the location information (and therefore presence of a local reservoir) to herds which have had a breakdown, it better reflects the known most important predictor. We have added text to this effect in the discussion.*

6. Why is it reasonable to neglect transmission within the reservoirs? This question should be discussed in the text.

*Response: The purpose of the model is to consider the ‘net’ effect of transmission via the generation of diversity plus the estimated transmission rate per reservoir location. Where transmission is low, generation of diversity and the transmission rate will likely be similarly low (if we assume that generation of diversity is strongly correlated with sustained transmission and circulation), and where it is high, generation of diversity is likely to be high. While clearly this involves substantial abstraction, the aim was to create an elegant model that incorporates this key element of the reservoir’s contribution to the observed phylogeny, without adding to many additional model elements (and therefore latent or unobserved processes)*. *We have provided further text in the manuscript to make this clearer.*

7. How is the metropolis-Hasting algorithm set up? What is the objective function? (e.g. is it based on matching the pairwise distributions of SNP differences, or does it include the observe incidence?)

*Response: We have added to the description of the Metropolis_Hastings algorithm an additional sentence and explained in the text that we use it to accept those parameter vectors that maximise the likelihood given in equation 2.*

8. Does table 2 refer to the best-likelihood model (model 3)? Should be stated in the table header.

*Response: We have edited the caption for table 2 stating that the posterior values given are for the model with the highest AIC score. We have also added a table to the supplementary information with the posteriors for the other models for comparison as this reviewer suggests (point 12 below).*

9. Do the model compared through the AIC differ in the number of parameters? If so, how? If the vector theta of parameters only include the parameters listed in the tables 1 and 2, then all models have the same number of parameters, and comparing AIC´s means comparing likelihoods.

*Response: All models have the same number of parameters so the AIC scores are indeed a comparison of likelihoods as reviewer points out (as the reviewer points out). As we expect that referring to the comparison in terms of AIC scores is familiar to most readers, we have kept this but added a short note explaining that we are, in effect, comparing likelihoods.*

10. How is the external force of infection obtained? Is there an statistical test that can support the claim that “R0 decreases slowly with increasing external force of infection”?, read in lines 253-254? (If not, that claim should be removed). I believe that the discussion around figure 3 is remarkably insufficient, and that this figure requires more explanation (I have to admit that I could not get to understand what is the point the autors try to make here). From a data vis. Point of view, very elementary questions are pertienent: what do points represent? Individual simulations, perhaps? How many dots are under R0=1?, What does the blue dashed line represent (the mean r0, perhaps?). I feel that, if the point here is to interrogate for the ability of the model to produce observed incidence levels, it would be desirable to also have a figure where observed vs simulated incidence is compared (see comment 12 below).

*Response: The purpose of the figure was to show that, in the absence of testing, the cattle only R0 is greater than one. Upon reflection over the reviewer’s comment, we agree that the figure is secondary and have shifted it to the supplementary information, also providing more text to emphasize that it is the value of R0 in the absence of the reservoir.*

11. In the parenthesis at the end of line 256 there is a typo.

*Response: this typo has now been fixed (the greater than sign wasn’t rendered by LaTeX)*

12. In figure 2 and table 2, authors present the results of their best-fit model. I think that, to be sure that the model 3 is really the best one (and that it reproduces coherently the data observed), there is information that is still missing. In my opinion, it would be important to include information about how different the predictions (e.g. likelihoods, observed vs simulated incidences, and pairwise distribution of SNPs differences) compare across the five models studied.

*Response: We have added a table (table 4) that compares the means of the posterior values for the models. While these posterior values are quite similar, it is more difficult of course to properly capture the characteristics of the full multi-dimensional posterior. To show that they are different, we follow the reviewer’s recommendation by showing the pairwise distribution of SNP differences. This is clearly sufficiently different when comparing between models to show that one is clearly favoured. This is now in a new figure in the main text (figure 4) comparing the data to the best fit of the various rejected models.*
